# Left Ventricular Fibrosis and CMR Tissue Characterization of Papillary Muscles in Mitral Valve Prolapse Patients

**DOI:** 10.21203/rs.3.rs-2936590/v1

**Published:** 2023-05-17

**Authors:** Ricardo A. Spampinato, Mateo Marin-Cuartas, Antonia Kampen, Florian Fahr, Franz Sieg, Elfriede Strotdrees, Cosima Jahnke, Kristin Klaeske, Karoline Wiesner, Jordan E. Morningstar, Yasufumi Nagata, David Izquierdo-Garcia, Maja-Theresa Dieterlen, Russell A. Norris, Robert A. Levine, Ingo Paetsch, Michael A. Borger

**Affiliations:** University, Leipzig Heart Center; University, Leipzig Heart Center; Harvard Medical School; University, Leipzig Heart Center; University, Leipzig Heart Center; University, Leipzig Heart Center; University, Leipzig Heart Center; University, Leipzig Heart Center; University, Leipzig Heart Center; Medical University of South Carolina; Harvard Medical School; Harvard Medical School; University, Leipzig Heart Center; Medical University of South Carolina; Harvard Medical School; University, Leipzig Heart Center; University, Leipzig Heart Center

**Keywords:** Mitral valve prolapse, myocardial fibrosis, cardiovascular magnetic resonance, dark-blood LGE, T1-mapping, papillary muscle

## Abstract

**Purpose.:**

Mitral valve prolapse (MVP) is associated with left ventricle (LV) fibrosis, including the papillary muscles (PM), which is in turn linked to malignant arrhythmias. This study aims to evaluate comprehensive tissue characterization of the PM by cardiovascular magnetic resonance (CMR) imaging and its association with LV fibrosis observed by intraoperative biopsies.

**Methods.:**

MVP patients with indication for surgery due to severe mitral regurgitation (n=19) underwent a preoperative CMR with characterization of the PM: dark-appearance on cine, T1 mapping, conventional bright blood (BB) and dark blood (DB) late gadolinium enhancement (LGE). CMR T1 mapping was performed on 21 healthy volunteers as controls. LV inferobasal myocardial biopsies were obtained in MVP patients and compared to CMR findings.

**Results.:**

MVP patients (54±10 years old, 14 male) had a dark-appearance of the PM with higher native T1 and extracellular volume (ECV) values compared with healthy volunteers (1096±78ms vs 994±54ms and 33.9±5.6% vs 25.9±3.1%, respectively, p<0.001). Seventeen MVP patients (89.5%) had fibrosis by biopsy. BB-LGE+ in LV and PM was identified in 5 (26.3%) patients, while DB-LGE+ was observed in LV in 9 (47.4%) and in PM in 15 (78.9%) patients. DB-LGE+ in PM was the only technique that showed no difference with detection of LV fibrosis by biopsy. Posteromedial PM was more frequently affected than the anterolateral (73.7% vs 36.8%, p=0.039) and correlated with biopsy-proven LV fibrosis (Rho 0.529, p=0.029).

**Conclusions.:**

CMR imaging in MVP patients referred for surgery shows a dark-appearance of the PM with higher T1 and ECV values compared with healthy volunteers. The presence of a positive DB-LGE at the posteromedial PM by CMR may serve as a better predictor of biopsy-proven LV inferobasal fibrosis than conventional CMR techniques.

## Introduction

Mitral valve prolapse (MVP) is the most common valvular heart disease in high-income countries with a prevalence of 2–3% (1). MVP may lead to ventricular arrhythmias and sudden cardiac death (SCD), mitral regurgitation (MR), left ventricular (LV) remodeling, and heart failure (1). Given the negative prognostic impact of myocardial changes secondary to MVP, increasing interest has been focused on early identification of LV structural alterations.

In MVP patients, regional myocardial fibrosis, mainly in the basal inferolateral LV wall (i.e. inferobasal region) and at the level of the papillary muscles (PM), has been demonstrated (2–6). This regional fibrosis has been hypothesized to be in part secondary to abnormal PM traction / mechanical stress in response to increased chordal tension from a prolapsing MV (4, 7–9). Importantly, recent data indicate that PM fibrosis may act as an arrhythmogenic substrate in MVP patients (2, 10, 11).

While conventional “bright-blood” LGE (BB-LGE) has been commonly used to detect PM fibrosis in MVP patients (2, 5, 12–14), more recently, Van De Heyning et al. showed that “dark-blood” LGE (DB-LGE) sequences with suppression of the blood pool signal were more sensitive for the detection of PM fibrosis (15). Furthermore, Scatteia et al. found a dark-appearance of the PM on cine images to be a typical feature of MVP (16). Finally, CMR T1 mapping is suitable to quantify diffuse interstitial fibrosis by increased extracellular volume (ECV) expansion and higher native T1 times (17–19), but has not been described at the level of the PM.

Hence, the aims of this prospective study involving MVP patients referred for MV surgery due to severe MR are: 1) to perform a comprehensive tissue characterization of the PM on CMR using healthy individuals as reference group for CMR T1 mapping-derived measurements; 2) to evaluate the diagnostic accuracy of conventional and DB-LGE compared to the results of intraoperative LV myocardial biopsy; and 3) to analyze the association between histological quantification of LV fibrosis and the comprehensive tissue characterization of the PM on CMR. We hypothesize that CMR tissue characterization of PM increases the identification of myocardial fibrosis in MVP patients as determined by myocardial biopsy.

## Material and Methods

### Study Population

Patients were prospectively enrolled in the study between March 2020 and February 2022. The study was conducted in accordance with the Declaration of Helsinki, and was approved by the local research ethics committee (450–18-ek). All subjects gave written informed consent prior to study initiation. Patients with MVP referred for MV surgery were included in the analysis. Screening and assessment of MVP and MR severity were performed using echocardiography according to standard guidelines (20). Exclusion criteria were atrial fibrillation, previous heart valve surgery, more than mild disease of other heart valves, intracardiac shunts, other known causes of cardiomyopathy including coronary artery disease, or typical contraindications for CMR imaging. All patients underwent successful MV repair via a right mini-thoracotomy approach.

Patient demographics and clinical data were obtained at the time of hospital admission for MV repair surgery, followed by echocardiography and CMR including a comprehensive tissue characterization of the PM.

Healthy individuals without MVP, more than mild heart valve disease, diabetes, and LV hypertrophy or other known cardiomyopathies were enrolled as a control group for CMR T1 mapping values.

### Echocardiography

Transthoracic echocardiography (TTE) was performed using standard commercially available ultrasound machines equipped with 2.25–4.25 MHz transducers. Evaluation of MVP was carried out by one cardiologist, expert in echocardiography and valvular heart diseases, blinded to the results of CMR exams. TTE was acquired using standard views, and Doppler measurements were evaluated as the average of three cycles. Color Doppler interrogation of the MR jet was performed in multiple views. A multimodal approach was used to determine MR severity, in concordance with recent guidelines (20, 21).

### Cardiovascular magnetic resonance imaging

All CMR examinations were performed on a dedicated 1.5 Tesla magnetic resonance scanner (Ingenia, Philips Healthcare, Best, The Netherlands) using a 28-element array coil. Image data acquisition (Additional file, Appendix) adhered to current recommendations (22). In brief, for cine CMR, steady-state free precession (SSFP) sequences were used during repetitive breath-holding. All standard cardiac geometries were acquired (multiple, gapless short-axis slices covering the entire left ventricle and 2-, 3- and 4-chamber views). Two-dimensional phase-contrast flow measurements were performed in the ascending aorta. Cine short axis images were used to measure left ventricular end-diastolic (LVEDV), end-systolic volume (LVESV), and left ventricular stroke volume (LVSV). With standard volumetric CMR regurgitant volume (RVol) and fraction (RF) were derived as previous described (23).

- *Papillary muscles signal (dark-appearance)*. For the measurement of PM signal, a mid-ventricular short-axis cine SSFP image in end-systolic phase was selected as it allowed for a better visualization of both PM. The PM signal was visually categorized as “dark- appearance” or “normal PM” when compared with LV myocardium, and quantified using the signal intensity ratio as previously described (16).
- *Late gadolinium enhancement*. Both BB-LGE and DB-LGE were performed in succession 10–15 min after the application of intravenous contrast (gadolinium-DTPA, 0.2 mmol/kg) in the same three long-axis and short-axis views used for cine imaging (Additional file, Appendix). All LGE images were acquired during breath-holding, and dark-blood was acquired as the last sequence of each CMR study.

Images were evaluated offi ine for the presence (LGE+) or absence (LGE-) of LGE in LV and both PM, LV pattern (subendocardial, mid-myocardial, subepicardial, transmural), and regional distribution of LGE areas using a standardized myocardial 17-segment model.

- *T1 and extracellular volume measurements*. An ECG-gated modified Look-Locker inversion (MOLLI) sequence with SSFP image readout was acquired at a basal, mid, and apical LV short-axis geometry pre- and 15 min post-contrast, making efforts to obtain the maximal myocardial mass of PM. Native T1 and ECV values were determined for the 16 LV segments of the short axis and separately also for both PM: anterolateral (PMal) and posteromedial (PMpm).

Post-processing was carried out offi ine (using IntelliSpace Portal 6, Philips Healthcare) with all readers fully blinded to clinical and echocardiographic data. T1-mapping, BB-LGE and DB-LGE sequences were analyzed by a cardiologist (RS) with over 7 years of experience in CMR imaging, separated and in random order at least 4 weeks apart from initial acquisitions, and blinded to the general imaging and histological data. In case of uncertainty, consensus was provided by a second level III observer (CJ).

#### Histological and immunohistochemical evaluation

LV biopsies were obtained from each patient from the inferobasal LV wall using a surgical rongeur under thoracoscopic guidance. The biopsies were fixed in 4% formaldehyde/phosphate-buffered saline and embedded in paraffi n before cutting 3 μm sections. Histopathology and immunohistochemistry methods are described in the Appendix (Additional file). Briefly, Masson-Goldner’s trichrome staining kit (Carl Roth GmbH, Karlsruhe, Germany) was used to stain connective tissue and collagen I was stained by immunohistochemistry using the mouse anti-collagen I antibody (Abcam, Cambridge, UK).

Documentation and quantification of connective tissue and collagen were performed using AxioPlan 2 microscope, Axio Cam camera system, AxioVision Release 4.8.2 SP3 software (Carl Zeiss, Jena, Germany), and Adobe Photoshop CS2 software (Adobe Inc., Mountain View, CA, USA). Fibrosis burden was quantified as connective tissue volume fraction (CVF) and a normal upper limit of 10.9%, as previously reported, was used to dichotomize into normal/abnormal (24).

#### Statistical Analysis

Normal distribution was assessed with Q-Q plots and Shapiro-Wilk test. Data are presented as mean and standard deviations for normally distributed continuous variables, median and interquartile range (25^th^ to 75^th^ percentile) for non-normally distributed continuous variables, or frequency and percentages for categorical variables. Statistical differences between groups were assessed using Student’s t-test for continuous variables or Fisher’s exact test for categorical variables. Differences in the detection of fibrosis between histology, conventional bright-blood and dark-blood modalities were evaluated using McNemar’s test. Spearman or Pearson (for linear relationships) coeffi cient was used to assess correlations between variables. Two-tailed p-values <0.05 were considered statistically significant. Analyses were performed using SPSS software (IBM-SPSS Statistics, Version 20, IBM Corp.).

## Results

A total of 19 MVP patients (mean age 54 ±11 years, 74 % male) were enrolled in the study and underwent CMR imaging preoperatively and myocardial biopsy during MV surgery. A group of 21 healthy volunteers (mean age 49 ±14 years, 52 % male) also underwent a 1.5T CMR scan including T1 mapping of the PM for comparison. CMR T1 mapping analysis was successfully performed in all PM of MVP patients and in 90.5 % of the PM in the control group (4 of 42 PM were too small to be depicted with a ROI).

Demographic and baseline patient characteristics are presented in Table 1. There were no statistical differences between MVP patients and healthy volunteers for any variables, with the exception of NYHA classification which was class I in all control patients. CMR and TTE characteristics are shown in Table 2. Patients with MVP had high RVol and RF values in accordance with severe MR, and consequently higher CMR-derived LVEDV and LVESV compared with controls. However, LV systolic function and natriuretic peptide values (Table 1) were within the normal range in MVP patients.

### Tissue characterization of PM

**Figure 1:**
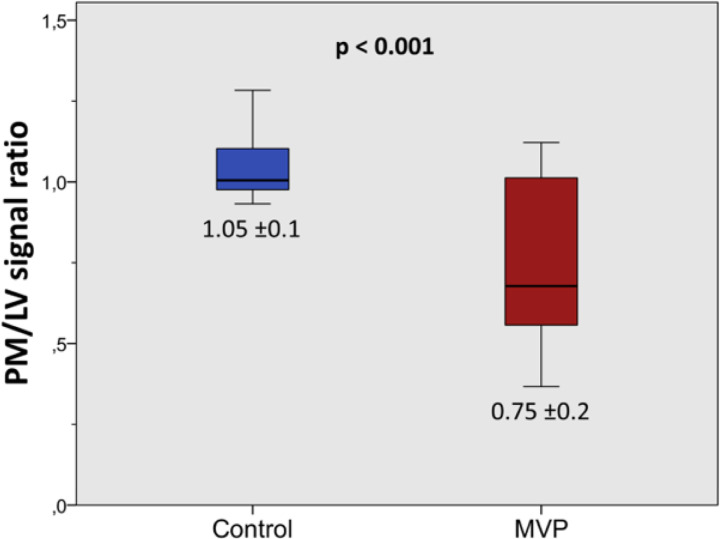
Dark-appearance of the papillary muscles (PM). Signal intensity ratio between PM and left ventricle (LV) showing a lower ratio (dark-appearance) of the PM in mitral valve prolapse (MVP) patients but not in healthy volunteers.

**Figure 2:**
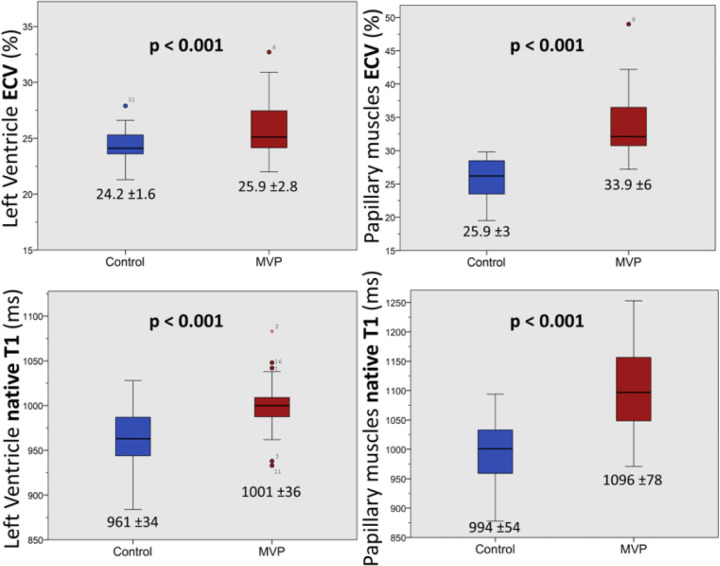
Left ventricle and papillary muscles extracellular volume (ECV) and native T1. Mitral valve prolapse (MVP) patients showed higher CMR T1 mapping parameters values compared with healthy volunteers.

**Figure 3:**
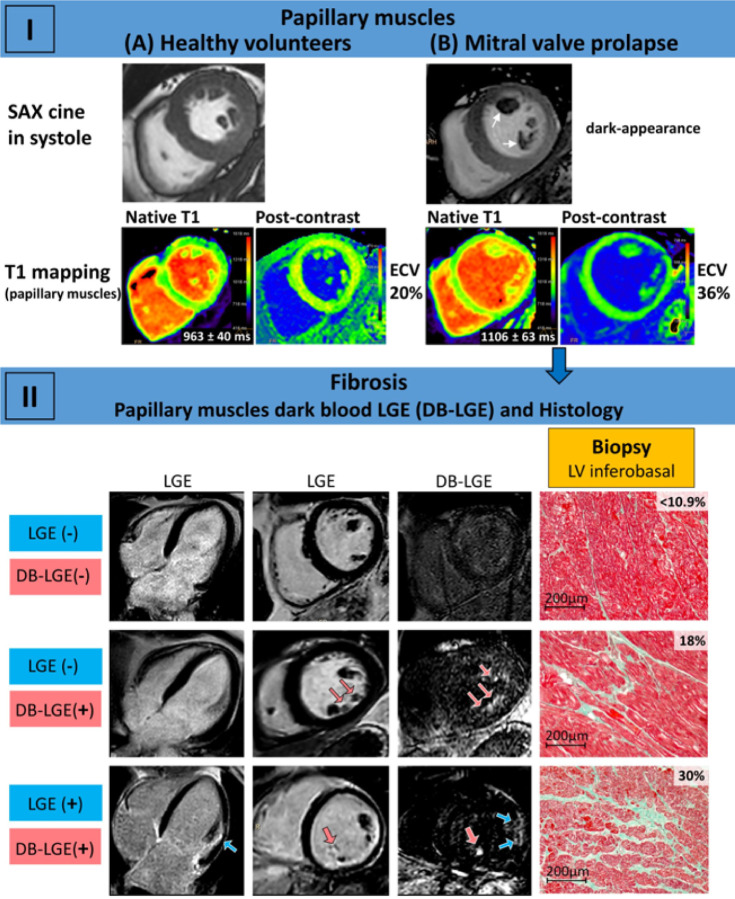
CMR Tissue Characterization of Papillary Muscles (PM) in Mitral Valve Prolapse (MVP) Patients. Part I; CMR short axis cine, and native and post- contrast T1 mapping depicting characteristic higher extracellular volume (ECV) and native T1 values of the PM in MVP patients (B) compared with healthy volunteers (A). White arrows show the dark-appearance of the PM. Part II; in MVP patients, while the presence of a positive “dark blood” late gadolinium enhancement (DB-LGE+) at the posteromedial PM by CMR correlated with biopsy proven left ventricle (LV) fibrosis of the inferobasal wall, none of the others CMR parameters do so, including conventional LGE. Red arrows show replacement fibrosis of PM and blue ones of LV wall as demonstrated by LGE. Exemplary Masson-Goldner’s trichrome staining for visualization of connective tissue fraction are depicted (normal upper limit 10.9 %). Scale bars = 200μm.

In the CMR derived SAX cine, MVP patients had a significant lower PM/LV signal ratio compared with healthy volunteers (Figure 1) showing a dark-appearance of the PM in 13 cases (68.5 %). Moreover, MVP patients had higher LV native T1 (1001 ±36ms vs. 961 ±34ms, p = 0.001) and ECV (25.9 ±2.8% vs. 24.2 ±1.6%, p = 0.025) values than controls, as well as higher PM native T1 (1096 ±78ms vs. 994 ±54ms, p <0.001) and ECV (33.9 ±5.6% vs. 25.9 ±3.1%, p <0.001) values (Figure 2 and Figure 3, part I).

### CMR assessment of myocardial fibrosis

LV myocardial LGE was present in 5 of 19 MVP subjects by BB-LGE and in 9 subjects by DB-LGE (26.3 % vs. 47.4 %, p = 0.063). By BB-LGE intra-myocardial pattern was present in 2 cases and a sub-endocardial pattern in 3 cases, with location in basal inferior (n = 3), basal inferolateral (n = 3), basal anterolateral (n = 1), mid inferoseptal (n = 1), mid inferior (n = 2), or mid inferolateral (n = 3) LV segments. By DB-LGE intra-myocardial pattern was present in 2 cases and a sub-endocardial pattern in 7 cases, located in the basal inferoseptal (n = 1), basal inferior (n = 3), basal inferolateral (n = 4), basal anterolateral (n = 1), mid inferoseptal (n = 2), mid inferior (n = 5), mid inferolateral (n = 4), apical inferior (n = 1), or apical lateral (n = 1) LV segments. There was insuffi cient evidence to support differences in the numbers of LV segments detected by each LGE methods.

LGE was detected in the PM in 5 subjects by BB-LGE and in 15 subjects by DB-LGE (26.3 % vs. 78.9 %, p = 0.001). All cases with BB-LGE+ at the level of the PM were confirmed on DB-LGE. Overall, fibrosis was found more frequently in the PMpm than the PMal (73.7 % vs. 36.8 %, p = 0.039).

### Biopsy derived myocardial fibrosis and correlations with CMR parameters

In all MVP patients, a myocardial biopsy was obtained from the inferobasal LV region. The extent of histological fibrosis was quantifed by CVF with a mean value of 25.1 ±16% and a collagen I fraction of 13.7 ±11%. In the entire cohort, a total of 17 MVP patients (89.5 %) had an abnormal histological finding (CVF >10.9 %). There were no sex-related differences in fibrosis (CVF male 22.8 ±15% vs. female 31.2 ±17%, p = 0.369).

**Figure 4:**
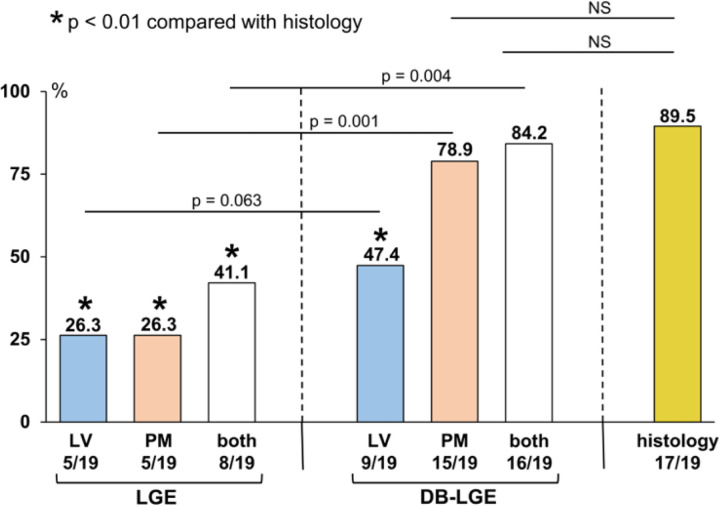
Detection of fibrosis with CMR and histology, LGE, late gadolinium enhancement; DB; dark blood; LV, left ventricle; NS, no significant; PM, papillary muscles.

**Figure 5:**
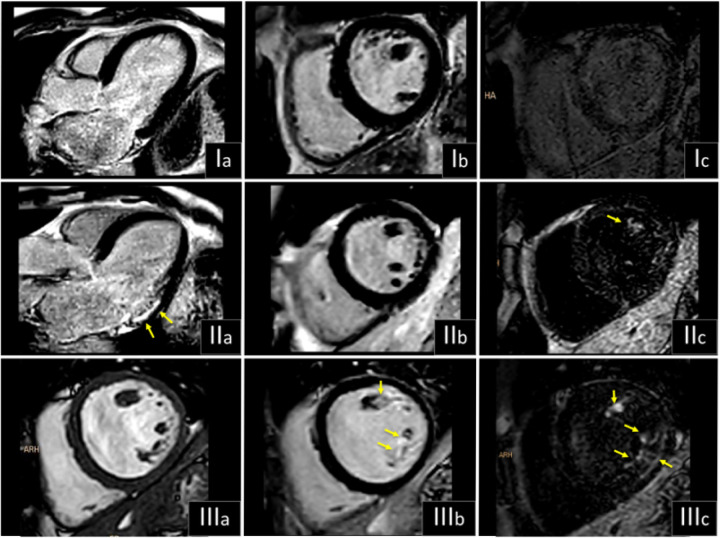
Examples of three MVP patient. I, without late gadolinium enhancement (LGE); II and III, with positive LGE: (b) conventional “bright blood” LGE (BB-LGE+) or (c) “dark blood” LGE (DB-LGE+). Ia, normal LV long axis BB-LGE; IIa, LV long axis depicting a subendocardial scar by BB-LGE; IIIa, LV short axis cine, note the loss of myocardial mass of the PM in image IIIb (BB-LGE+) confirmed in image IIIc (DB-LGE+). Arrows are depicting a positive LGE phenomenon.

Compared with histologic findings, only PM DB-LGE+ showed no statistical differences in detection of fibrosis (89.5 % vs. 78.9 %; Figure 4). Figure 5 shows three examples of MVP patients with and without LGE.

The extent of biopsy-derived CVF and the presence of DB-LGE+ at the PM had both a significant inverse correlation with CMR LV volumes, and CVF likewise with MR severity (Table 3). From all CMR parameters for PM tissue characterization, only a DB-LGE+ at the level of the PMpm was directly correlated with both biopsy derived parameters: CVF and collagen I fraction from the inferobasal LV region (Figure 3, part II). Finally, CMR derived PM ECV significantly correlated with the extent of mitral annular disjunction (MAD), with PM native T1 values, and with the presence of PM DB-LGE+, particularly at the PMpm (Table 3).

## Discussion

This is the first study of CMR derived tissue characterization of the PM combining CMR cine images, T1 mapping, conventional and DB-LGE sequences and comparing its results with histological assessment of myocardial fibrosis of the inferobasal LV wall in MVP patients.

The main findings of this study are: 1) only MVP patients showed a dark-appearance of the PM on CMR cine compared with healthy volunteers, with higher native T1 and ECV values at the level of the LV myocardium and even more pronounced at the level of the PM; 2) with the use of a DB-LGE technique there was only a tendency to detect a higher prevalence of LGE within LV myocardium, but a significantly higher rate of LGE detection at the level of the PM when compared with BB-LGE; 3) compared with histology, only PM DB-LGE showed no statistical differences in detection of fibrosis; 4) the presence of DB-LGE + at the PMpm correlated with biopsy-proven fibrosis of the inferobasal LV wall, while other CMR sequences did not; and 5) there was an inverse correlation between extent of fibrosis and CMR derived LV volumes and MR severity.

### CMR tissue characterization of the papillary muscles

Scatteia and coworkers have previously described a dark-appearance of the PM on standard short axis cine CMR images as a typical feature of MVP, which was not linked to myocardial fibrosis by LGE or to the occurrence of malignant arrhythmias (16). In accordance with their findings, we also failed to demonstrate a correlation between dark-appearance of the PM on CMR cine and PM T1 mapping parameters, with LGE at the level of the PM or LV myocardium, or with histologically-proven LV fibrosis. This might suggest that dark-appearance of the PM in CMR cine constitutes an early sign related to increased mechanical stress (9).

While altered T1 mapping parameters at the level of the LV in MVP patients (12, 19) and its association with worse remodeling after mitral valve repair (25) have been described by others, our study, to the best of our knowledge, is the first to describe higher native T1 and EVC values at the level of the PM in this population. The PM T1 mapping parameters were obtained in all MVP patients. PM ECV was associated with the presence of scar detected by DB-LGE within the PM, particularly the PMpm. However, PM ECV values did not show significant correlation with biopsy-derived CVF, but with extent of MAD. It could be hypothesized that an increased PM ECV might be a marker of increased annular disjunction or stretch of mitral valvular apparatus even before the onset of focal PM fibrosis on CMR or inferobasal LV wall fibrosis in histology. In fact, although patients with DB-LGE + at the level of the PM had significant higher ECV values (PM ECV: 35.7 ± 5.5% vs 29.1 ± 1.5%, p = 0.011), those without DB-LGE still showed elevated ECV values compared with healthy volunteers (PM ECV: 29.1 ± 1.5% vs 25.9 ± 3.0%, p = 0.005; upper interquartile range in healthy volunteers: 28.6%).

### Detection of myocardial fibrosis

With BB-LGE, we detected LV myocardial late contrast uptake in 5/19 patients (26%) with MVP and severe MR, similar to previous studies demonstrating (mostly inferobasal) LV myocardial LGE in around 25 to 37% of MVP patients (5, 6, 13, 26, 27), paralleling recent findings of our group in human patients and genetically modified mice with MVP (4). This regional LV fibrosis has been found to be more prevalent in patients with MR and MVP than in patients with MR and no MVP (3, 5, 6), supporting a more direct relationship between MVP and early LV fibrosis. Indeed, there is evidence of myocardial fibrosis before the onset of symptoms or macroscopically obvious LV structural changes in patients with primary MR (26).

We could not find a significant difference between BB-LGE and DB-LGE in detecting LV myocardial surrogate of fibrosis (26.3% vs. 47.4%, p = 0.063). However, we observed a higher prevalence of LGE + at the level of the PM with DB-LGE compared with BB-LGE (78.9% vs. 26.3%, p = 0.001). Previous studies using conventional BB-LGE technique reported PM LGE in around 7 to 36% (5, 12, 13, 15, 26). DB-LGE technique increases the scar-to-blood contrast by suppressing the blood pool signal, which might improve the myocardial scar conspicuity and, thus be more sensitive for detection of PM fibrosis. In line with our data, a previous study (15) also demonstrated that compared to BB-LGE, DB-LGE improves the detection of LGE at the PM (15% vs 35%) – but that study did not include histological analysis. Previous reports showed surrogate of PM fibrosis in up to 63% of MVP patients with life-threatening ventricular arrhythmias (2, 14). Recent data indicate that PM fibrosis may act as an arrhythmogenic substrate in these patients (10, 28). We found an abnormal histology of the inferobasal LV wall in 17/19 patients, without statistical differences in detection of fibrosis with DBLGE (89.5% vs. 78.9%; Fig. 4). Further supporting the arrythmogenic role of fibrosis in MVP, Basso et al. observed LV scarring at the level of PM in all and of the inferobasal LV wall in 88% of selected young SCD victims with MVP (2).

Interestingly, we found that the PMpm was affected more frequently than the PMal and we describe for the first time a significant correlation between DB-LGE + at the PMpm and fibrosis by biopsy of the inferobasal LV wall. Previous studies demonstrated less contraction or higher stretch of the PM in MVP patients(8). In a CMR study, Han et al. described a higher excursion of the PMpm tip towards the mitral annular plane during systole in MVP patients compared with healthy volunteers; yet, did not compare it with the PMal (29). A more recent echocardiography study (30) found significantly lower longitudinal strain (i.e. less shortening) of the PMpm in MVP patients compared with controls (−30.5% vs. −43%), with no differences regarding the PMal, suggesting more pronounced mechanical stress of the posteromedial than the anterolateral PM.

We did not find a correlation between LV LGE and inferobasal LV myocardial biopsy. This is in line with findings by Liu and colleagues (26), and might suggest an underestimation of fibrosis even with the use of DB-LGE at the level of LV myocardium.

Finally, we found an inverse correlation between CVF by biopsy and CMR LV volumes and MR severity (Table 3). Although, there is evidence demonstrating presence of LV fibrosis in MVP patients with less MR (31), previous reports also showed that MVP patients with LV LGE had more severe MR (25). The Jensen group (32), using a novel vacuum-based ex-vivo model, showed that the total PM force varies linearly with the trans-mitral pressure (i.e. gradient between LV and left atrial pressure), and numerically more pronounced at the medial PM. Theoretically, more severe MR increases the LA pressure, which leads to a decrease in trans-mitral pressure gradient, with consequently lower PM traction force. Thus, hypothetically, MVP patients with pronounced flail leaflet and higher RVol might have less PM stretch. Nevertheless, this remains a purely hypothetical explanation, as our study provides insuffi cient evidence to support this theory - not only because of the small number of subjects, but especially because of the cross-sectional study nature. We were not able to analyze if patients who had PM LGE had long periods of less severe MR or, inversely, if those without PM LGE developed a flail leaflet in an earlier phase of the disease. Moreover, LV volume overload in MVP has been associated with increased interstitial space but progressive collagen degradation (33). Loss of extracellular matrix may allow for a more compliant LV.

### Clinical implications

Regionalized inferobasal LV and PM fibrosis have been identified as an arrhythmogenic substrate and plausible explanation for SCD even in asymptomatic patients with hemodynamically uncomplicated MVP (2, 14, 34, 35). An increasing understanding of these mechanisms raises the question of whether surgical treatment or other medical interventions in a subgroup of high-risk selected MVP patients should occur earlier than currently indicated (36, 37). The major challenge is the early identification of patients at risk for developing severe ventricular arrhythmias or irreversible LV dysfunction within a large population of patients with asymptomatic hemodynamically uncomplicated MVP (33, 35). The proposed CMR tissue characterization of the PM may serve as a supplementary diagnostic tool with potential application in this area.

### Study limitations

The present study has some limitations. First, the study was not powered or designed to assess the relationship of the results with MVP subtypes or with clinical characteristics like ventricular arrhythmias. Second, the results should be interpreted with caution since a small sample size could lead to type II errors and correlations could not be adjusted for type I errors. Finally, histological findings might be limited by sampling error. Thus, further studies utilizing a longitudinal design are required to better evaluate the influence of MVP subtypes and disease progression on unfavorable myocardial structural changes and cardiac arrhythmias.

## Conclusions

CMR derived tissue characterization of the PM in MVP patients referred for MV surgery due to severe MR, demonstrates a characteristic CMR pattern of the PM with dark-appearance and higher T1 and ECV values compared with healthy volunteers and a possible better prediction of biopsy-proven LV inferobasal fibrosis when a positive DB-LGE at the posteromedial PM (PMpm) is present.

## Figures and Tables

**Table 1:** Baseline characteristics

	MVP (n = 19)	Controls (n = 21)
**Clinical characteristics**		
Age, years	54 ±10	49 ±14*
Male, n (%)	14 (74)	11 (52)*
BMI (kg/m^2^)	25 ±5	26 ±4*
BSA (m^2^)	1.98 ±0.2	1.96 ±0.3*
Systolic BP, mmHg	140 ±17	133 ±15*
Diastolic BP, mmHg	80 ±13	75 ±13*
CAD, n (%)	0	0
Hypertension, n (%)	10 (53)	8 (38)*
Diabetes, n (%)	0	0
Dyslipidemia, n (%)	4 (21)	5 (24)*
NYHA I/II/III-IV, n	7/11/1	21/0/0
eGFR (ml/min/1.73m^2^)	92.5 ±13	-
NTproBNP (ng/L)	148 (98 – 353)	-
**Mitral ValVe Lesion**		
Posterior leaflet, n	15	-
Anterior leaflet, n	4	-
Bileaflet (Barlow’s disease), n	4	-

**MVP**, mitral valve prolapse; **BMI**, body mass index; **BSA**, body surface area; **CAD**, coronary artery disease; **NYHA**, New York Heart Association Functional Classification; **eGFR**, estimated glomerular filtration rate; **NTproBNP**, N-terminal pro-brain natriuretic peptide. Data are reported as n (%), as mean±SD, or median (interquartile range).

* Variables show no statistical differences compared with MVP.

**Table 2.** Echocardiographic and Cardiac Magnetic Resonance (CMR) Values.

	Control (21)	MVP (19)	P-value
**Echocardiographic parameters**			
EDV, ml	-	197 ±56	n.a.
ESV ml	-	66 ±26	n.a.
EF, %	-	67 ±7	n.a.
LVOT SV ml	-	131 ±36	n.a.
RVol, ml *	-	73 ±39	n.a.
RF, % *	-	47 ±18	n.a.
**CMR parameters**			
EDV, ml	145 ±35	233 ±51	<0.001
ESV ml	57 ±18	87 ±20	<0.001
LV SV, ml	88 ±19	145 ±34	<0.001
EF, %	61 ±4	62 ±4	0.321
LA area, cm^2^	21 ±5	31 ±9	<0.001
Aorta net flow (SV), ml	80 ±17	76 ±15	0.534
RVol, ml	9 ±5	67 ±26	<0.001
RF, %	10 ±6	45 ±12	<0.001
Curling motion, n (%)	0	8 (42)	
MAD, n (%)	0	7 (37)	
**Tissue characterization**			
LV native T1, ms	961 ±34	1001 ±36	0.001
LV ECV, %	24.2 ±1.6	25.9 ±2.8	0.025
PM native T1, ms	994 ±54	1096 ±78	<0.001
PM ECV, %	25.9 ±3	33.9 ±6	<0.001
PM/LV_ratio	1.05 ±0.1	0.75 ±0.2	<0.001
PM dark appearance, n (%)	0	13 (68)	<0.001
LV LGE presence, n (%)	0	5 (26.3)	0.018

**MVP**, mitral valve prolapse; **EDV**, LV end-diastolicvolume; **ESV**, LV end-systolic volume; **EF**, left ventricular ejection fraction; **LVOT**, left ventricular outflow tract; **SV**, stroke volume; **RVol**, regurgitant volume (mitral valve); **RF**, regurgitant fraction (mitral valve); **LA**, left atrial; **LV**, left ventricle; **ECV**, extracellular volume; **PM**, papillary muscles; **LGE**, late gadolinium enhancement. Values are expressed as mean±SD.

**Table 3:** Correlation of DB-LGE of LV and PM, extracellular volume (ECV) of PM, and histology (CVF and Collagen I).

	LV BD-LGE		PM DB-LGE		PMal DB-LGE		PMpm DB-LGE		PM ECV		CVF, %		Collagen I, %	
	Rho	p-value	Rho	p-value	Rho	p-value	Rho	p-value	Rho	p-value	Rho	p-value	Rho	p-value
LV EDV ml	−0.241	0.320	−0.425	0.070	−0.150	0.541	−0.515	**0.024**	−0.343	0.151	−0.674	**0.002**	−0.098	0.691
LV ESV, ml	−0.087	0.724	−0.566	**0.012**	−0.469	**0.043**	−0.515	**0.024**	−0.414	0.078	−0.795	**<0.001**	−0.142	0.561
EF, %	−0.232	0.339	0.438	0.061	0.260	0.282	0.351	0.141	0.322	0.179	0.078	0.750	0.235	0.332
MR volume, ml	0.019	0.938	−0.141	0.564	0.139	0.569	−0.262	0.279	0.072	0.770	−0.649	**0.003**	−0.033	0.892
MR fraction, %	0.404	0.086	−0.024	0.924	0.299	0.214	−0.196	0.420	0.321	0.180	−0.616	**0.005**	−0.016	0.949
Curling motion*	0.258	0.285	0.179	0.464	0.012	0.962	0.268	0.268	0.370	0.119	0.272	0.259	0.311	0.194
MAD, mm	0.400	0.090	0.204	0.402	0.092	0.708	0.277	0.250	0.474	**0.040**	0.116	0.638	0.181	0.457
PAPs (mmHg)	0.212	0.384	0.248	0.307	0.588	**0.008**	0.142	0.562	0.134	0.584	−0.086	0.726	−0.391	0.098
NTproBNP	0.246	0.325	0.273	0.273	0.340	0.167	0.103	0.684	0.098	0.699	0.057	0.823	−0.265	0.287
Barlow’s*	0.286	0.236	−0.050	0.839	−0.394	0.095	0.015	0.950	0.212	0.383	0.189	0.439	0.283	0.241
LV native T1, ms	0.029	0.907	−0.342	0.152	−0.259	0.284	−0.404	0.086	−0.338	0.157	−0.354	0.137	−0.215	0.377
LV ECV, %	0.607	**0.006**	0.142	0.563	0.409	0.082	−0.044	0.859	0.462	**0.047**	0.170	0.486	−0.227	0.349
PM native T1, ms	0.231	0.341	0.495	**0.031**	0.100	0.685	0.665	**0.002**	0.721	**<0.001**	0.226	0.351	0.372	0.117
PM ECV %	0.346	0.146	0.589	**0.008**	0.438	0.061	0.665	**0.002**	-	-	0.400	0.090	0.340	0.154
PM dark*	0.191	0.434	0.141	0.565	0.049	0.841	0.108	0.659	0.279	0.248	0.040	0.871	0.103	0.674
LV LGE presence*	0.630	**0.004**	0.309	0.199	0.287	0.234	0.086	0.727	0.087	0.722	−0.218	0.369	−0.349	0.143
LV DB-LGE presence*	-	-	0.231	0.341	0.368	0.121	0.088	0.720	0.346	0.146	−0.115	0.638	−0.289	0.231
PM LGE presence*	0.391	0.098	0.309	0.199	0.039	0.874	0.086	0.727	−0.087	0.722	−0.327	0.171	−0.022	0.929
PM DB-LGE presence*	0.231	0.341	-	-	0.394	0.095	0.864	**<0.001**	0.589	**0.008**	0.354	0.138	0.330	0.168
PMal DB-LGE presence*	0.368	0.121	0.394	0.095	-	-	0.209	0.391	0.438	0.061	0.239	0.324	−0.080	0.746
PMpm DB-LGE presence*	0.088	0.720	0.864	**<0.001**	0.209	0.391	-	-	0.655	**0.002**	0.502	**0.029**	0.480	**0.038**
CVF, %	−0.115	0.638	0.354	0.138	0.329	0.324	0.529	**0.029**	0.400	0.090	-	-	0.244	0.314
Collagen I, %	−0.289	0.231	0.330	0.168	−0.080	0.746	0.480	**0.038**	0.340	0.154	0.244	0.314	-	-

Results are from (Rho) correlation coeffi cients. Bold P-values are significant at P<0.05. **LV**, left ventricle; **EDV**, end-diastolic volume; **ESV**, end- systolic volume; **EF**, left ventricular ejection fraction; **MR**, mitral regurgitation; **MAD**, mitral annulus disjunction; **PAPs**, pulmonary artery systolic pressure; **NTproBNP**, N-terminal pro-brain natriuretic peptide; **PM**, papillary muscles; **al**, anterolateral; **pm**, posteromedial; **LGE**, Late Gadolinium Enhancement; **DB**, dark blood; **CVF**, connective tissue volume fraction.

* Dichotomous variables.

## Abbreviations

BB: bright blood (conventional)
CMR: cardiovascular magnetic resonance
CVF: connective tissue volume fraction
DB: dark blood
ECV: extracellular volume
LGE: late gadolinium enhancement
LV: left ventricle
MVP: mitral valve prolapse
PM: papillary muscle
PMal: anterolateral papillary muscle
PMpm: posteromedial papillary muscle
